# Exploring Unmet Information Needs of People with Parkinson’s Disease and Their Families: Focusing on Information Sharing in an Online Patient Community

**DOI:** 10.3390/ijerph19052521

**Published:** 2022-02-22

**Authors:** Hyeon Sik Chu, Hye Young Jang

**Affiliations:** School of Nursing, Hanyang University, 222 Wangsimni-ro Seongdong-gu, Seoul 04763, Korea; healingchu@hanyang.ac.kr

**Keywords:** access to information, Parkinson disease, patient-centered care, caregivers, online community

## Abstract

This study aimed to examine the unmet information needs of people with Parkinson’s disease and their family members by analyzing Parkinson’s disease-related posts in online communities. Data were collected from one of the largest online people with Parkinson’s disease communities used in South Korea. The word cloud, the main questions from the free-posting messages, as well as the frequently asked symptoms and side effects of the medication, were analyzed using content analysis. The commonly mentioned main questions from the free-posting messages have pertained to treatment-related information, such as effects and side effects of medication, deep brain stimulation, and complementary and alternative medicine. People with Parkinson’s disease and their families depend not only on health care providers but also on using online communities to find the information that they need. However, there is a need for treatment-specific information, such as anti-Parkinson drugs, deep brain stimulation, and complementary alternative therapies. As for the method of providing information for people with Parkinson’s disease and their families, it will be effective to provide tailored education services using online communities and social media by using their information needs and preferred resources.

## 1. Introduction

Parkinson’s disease (PD) is a major neurodegenerative disease whose worldwide incidence has been on the rise, as the population ages [1]. As PD progresses, patients experience movement-related symptoms, such as tremors, bradykinesia, and postural instability, which limit independent activities of daily living performance and social activities [2]. In addition, people with PD experience non-motor symptoms, such as pain, sleep disturbances, constipation, fatigue, and depression that are common throughout all stages of PD, and as the prevalence period increases, they may experience more diverse and serious symptomatic problems [3,4,5]. Although PD is a complex disease that causes both physical and psychosocial health, along with a significant impact on people with PD and their family’s quality of life [6]. For people with PD, therapeutic approaches, such as medications, surgery, and exercise, have shown positive results in prolonging life expectancy, improving daily life adaptation, and raising the quality of life [7,8]. However, when a diagnosis is made, people with PD and their family members feel desperate and uncertain about the disease’s course due to the lack of a cure [6,9]. Therefore, it is necessary to derive appropriate measures for providing accurate information at the right time to help people cope with and adapt to the disease.

Online community-based self-help gatherings for people with specific diseases are actively utilized as places for information exchange, communication, and social interactions. Information exchange using the internet is actively employed [10]. As a result of improved technical possibilities, online communities for people with specific disease and their families have developed. Sharing experiences can help one understand their illness and compare their situations. They can learn from others, decide about health care, such as treatment options, and share knowledge via online communities [11]. Through interactions with each other, they not only share experiences and raise awareness of certain issues among themselves but also amongst healthcare providers and the research community [12]. In recent years, participation in online patient communities by patients and their families has become a subject of scientific investigation [10,11,12,13]. These studies conducted surveys using the user pool in the online communities or interactions between online community users on specific topics. Through these studies on the communities, it was identified that online communities have the potential to contribute to the quality of care by increasing patients and their family members’ empowerment [14].

Health care professionals must play a role in improving patients’ quality of life by providing services that are both patient and user centered, as well as support patients living with their families [15]. Thus, the specific information desired by people with PD and their families must be identified. Currently, only a few studies have been conducted on the experiences and needs of people with PD [16,17,18]. These previous studies emphasize the importance of providing tailored information at a right time for people with PD to make decisions and share information with family members, and for self-management. However, most of them did not include family members but instead focused only on the patients’ experiences and needs. Family members have the greatest influence on the patient’s condition, and the support of one’s family has been reported as an important factor in adapting to the disease [2]. Considering that as the disease progresses, the family has to play the role of a primary caregiver; it is necessary to examine the experiences and needs of not only the patients but also their families, which could be helpful in preparing adequate measures for improvement [6]. However, most of the previous studies with people with PD and their families have been conducted in a clinical setting with a small sample size and mainly in Western countries [18,19].

Therefore, this study aims to identify the content and characteristics of the information that people with PD and their family members desire by analyzing postings on an online patient community bulletin board. In this study, a word cloud was created to present frequently used terms related to PD using free-posting messages, and unmet information needs of people with PD and their families were identified using content analysis of the free-posting messages. In addition, the details of effects and side effects of medication and PD-related symptoms, which were the highest unmet information needs, were classified. Its results can be used as a basis for developing educational materials and nursing intervention programs for people with PD and their families.

## 2. Methods

### 2.1. Data and Ethical Considerations

This study was conducted with data from one of the largest online communities used by people with PD and their family members in South Korea via “NAVER” (https://cafe.naver.com/parkinson777; accessed on 5 August 2019). With nearly 11,844 members, this website serves as an online space for its members—people with PD and their families—to share a variety of information on the disease and social support.

After obtaining permission from an operator of the online people with PD community, data were collected from the messages that had been posted on the bulletin board from July 2017 to July 2019. When collecting data, we excluded personally identifiable information, including ID, name, and residential area from the contents of free postings. While 1378 messages were collated using an Excel 2016 spreadsheet, after excluding 30 messages that contained commercial advertisements or were unrelated to PD, a total of 1348 posted free-texts were analyzed for this study, which was approved by the institutional review board of the institution to which the researcher belongs (IRB No. HYU-2019-07-0101).

### 2.2. Data Analysis

A content analysis was used for this research. First, the word “frequency” was analyzed, and the most frequently used words were extracted using the word frequency function of the Korean Natural Language Application (KoALA; https://www.koala4text.com; accessed on 20 April 2020). These words were then ranked in descending order after considering Korean word variations and we then made a word cloud. Second, the main questions of the free-posting messages were coded and categorized according to the classification scheme developed by Rutten et al. [20]. Finally, the frequency of commonly asked medication side effects was analyzed and frequently asked symptoms related to PD were categorized as motor or non-motor symptoms.

### 2.3. Inter-Rater Reliability Evaluation of the Coding System

Two coders identified word variations, such as analogous terms, spelling variations, and synonyms from a total of 1348 questions. Terms with word variations were treated as synonyms based on an agreement between the two coders (for example, enteral feeding, and enteral nutrition). The reliability and agreement between inter-coders were assessed using Cohen’s kappa coefficient.

## 3. Results

### 3.1. Inter-Rater Reliability

Cohen’s kappa coefficient is a statistic that is used to measure inter-rater reliability between two coders for categorical items. A Cohen’s Kappa value of 0.41–0.60 was considered as moderate agreement, more than 0.61–0.80 as substantial agreement, and more than 0.81 as almost perfect agreement [21]. When classifying unmet needs related to PD from free-posting messages, each coder independently classified categories and subcategories and calculated Cohen’s kappa coefficient. In this study, Cohen’s kappa was 0.86, with 90% agreement.

### 3.2. The Most Frequently Used Terms

**Figure 1:**
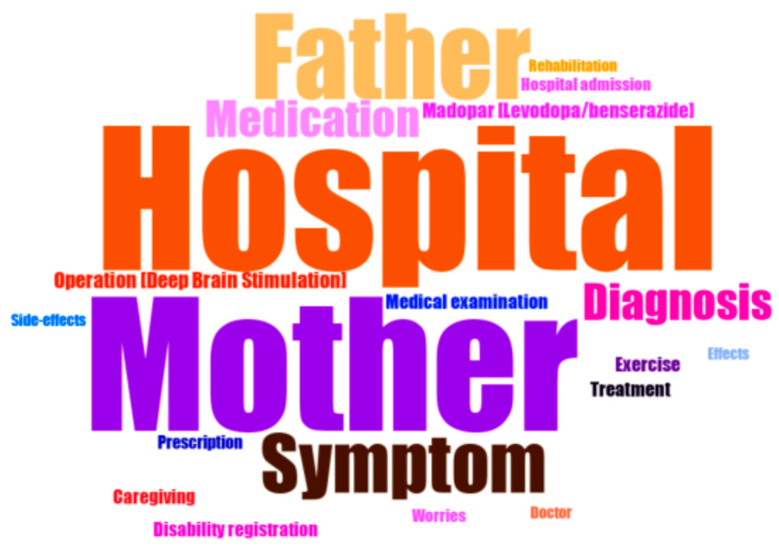
Word clouds of the frequently presented word in questions.

Word cloud (Figure 1) shows the 20 most frequently used terms related to PD. The font size of each word is determined by the word frequency. The higher the frequency terms appear to be, the bigger the font size. “Hospital”, which appeared in 1093 out of 1348 posts, was the most frequently used term. Frequently used words were “mother”, “father”, “Symptom”, “Diagnosis”, “Medication”, “Deep brain stimulation”, “Madopar^®^ (Levodopa/benserazide)”, and “Effect and side effect.”

### 3.3. Classification of Unmet Information Needs Related to PD from the Free-Posting Messages

We categorized the main questions from the free-posting messages into 7 main categories and 39 subcategories. Table 1 shows the typology of unmet information needs related to PD. The percentage of each category and subcategory was determined by dividing the frequency of the questions by 1348. The most frequently asked information was treatment-related information (41.01%), followed by Parkinson’s disease-specific information (30.57%), medical system information (12.54%), rehabilitation information (6.54%), interpersonal/social information (5.27%), care-related information (3.18%), and financial/legal information (0.96%). The five most frequently occurring subcategories were medication effects and side effects (20.18%), symptoms/management of symptoms (19.73%), hospital recommendations for treatment (5.86%), etiology and nature of the disease (5.34%), and complementary and alternative medicine (4.82%).

### 3.4. Frequently Asked Side Effects of Medication

We analyzed in detail the medication side effects, which are the most frequently asked in subcategories (Table 2). A total of 24 side effects of medications were asked, the most frequently asked medication side effects were hallucinations (17.09%), followed by drowsiness (14.53%), dizziness (11.97%), and nausea and vomiting (10.26%).

### 3.5. Frequently Asked Symptoms Related PD

The frequently asked symptoms related to PD were categorized as motor or non-motor symptoms. Almost two-thirds (64.66%) were categorized as non-motor symptoms. While the most frequently asked motor symptoms and non-motor symptoms were rigidity (8.65%), dysphagia (8.65%), and gastrointestinal symptoms (9.02%), respectively (Table 3).

## 4. Discussion

This study aimed to examine the information needs of people with PD and their families by analyzing PD-related posts in online communities. PD is a chronic neurodegenerative disease that leads to a person becoming more dependent on their family caregiver as the disease progresses. Family caregivers experience uncertainty while caring for patients and seek the information they need from online communities that have no time or space constraints [6]. Hence, health information is accessible anytime and anywhere, which is more advantageous for patients’ adult children who have multiple roles, not only family caregiver roles. In this regard, the online community can be a more extensive support system for adult children than the offline support system. Therefore, it is necessary to establish plans to facilitate online community involvement. It is also recommended to consider having a system that would use healthcare volunteers to answer patients’ questions and provide more reliable information.

In the word cloud, the most frequently appeared words were “hospital”, “mother” and “father”, respectively. This implies that most of the posts were made by people with PD children. Frequently used words were also related to diagnosis and treatment. It could be inferred that the people participating in the online community were people with PD and their families and were at various disease stages. While these words were similar to the frequently used words found by Park and Park [11], who analyzed online communities for people with cancer, the present study also found a word frequency that reflected the characteristics of treatments for PD, such as medication and surgical treatment. Through the word cloud of this study, it was found that people with PD and their families mainly try to supplement medical information among unmet information needs through the online community.

In this study, the most popular topic was treatment-related information. In particular, information on medication effects and side effects had the highest number. These results were in line with a study by Parsons and McGrath [22] that showed a high need for information about the time of inquiries relating to medication effects and side effects of people with PD and their family members. In particular, there were 117 posts that accounted for experiences of symptoms related to various medication side effects, the most frequent of which were hallucinations, drowsiness, dizziness, nausea, and vomiting; that are the known side effects of levodopa, dopamine agonists, and catechol-O-methyl transferase inhibitors, which are commonly used to treat PD [23]. In addition, there appeared a high need for information about the expected effects and side effects of medications. However, the questioners directed their queries on the effects and side effects of medications not to the healthcare professionals in charge of their treatment and care, but to the online communities. Thus, people with PD gathered information about the medications on their own and tried to find the right medication for themselves, which was considered helpful in improving their understanding of PD and self-care capacity [19,22]. However, especially for medication-related issues, people with PD should consult healthcare providers so as to maximize the effectiveness of the right medication for their conditions. Therefore, people with PD and their family members should be communicating their concerns in daily life and consult their healthcare providers during appointments. The healthcare professionals should provide a sufficient explanation about the drugs and their side effects, understand the patients’ concerns, and build a relationship with adequate emotional support. Healthcare professionals in charge of PD treatment and care need to provide sufficient explanations to people with PD and their families about medication effects and side effects, for which they can make use of labor resources, such as PD nurse specialists operating in the UK and US, who can help satisfy supportive care needs and improve the quality of life for people with PD and their families [1].

Moreover, as there is no cure, the need for information about deep brain stimulation—a surgical treatment as opposed to standard medical treatment—is high. Deep brain stimulation on advanced people with PD reduces motor symptoms, such as tremors and side effects caused by medication, by lowering the dose of prescribed medications [24]. However, people with PD and their families seem to face difficulty in acquiring information about the procedures, effects, and side effects of deep brain stimulation [25]. Hence, tailored education based on the stage of the disease seems necessary.

In this study, complementary and alternative medicine claimed third-place among treatment-related information needs. Similar results were shown in a study, in which 76.4% of people with PD reported receiving complementary and alternative therapies [26]. Some complementary and alternative therapies could have side effects and be more harmful to patients with severe illnesses [27]. Due to the large number of people with PD that use complementary and alternative therapies, healthcare professionals provide evidence-based information about the expected effects and side effects.

The second most popular topic was PD-specific information. In particular, among posts related to symptoms and their management, there were more questions on non-motor symptoms than motor symptoms. Motor symptoms, as well as non-motor symptoms, are factors that have a significant influence on people with PD’s quality of life. The most frequently reported non-motor symptoms in a study by Martinez-Martin et al. [28] were night urination, fatigue, and excessive salivation, which differ from our findings in which hallucinations, drowsiness, dizziness, nausea, and vomiting were the most frequent non-motor symptoms. This might have been due to the perceived differences in the severity of non-motor symptoms between people with PD and their family members. While people with PD and their family members can easily recognize motor symptoms rather than non-motor symptoms; however, as motor symptoms, such as postural instability and gait disturbance, show relationships with non-motor symptoms, such as fatigue and depression, people with PD and their family members need to understand both types of symptoms [3]. According to a study [29], providing people with PD with information customized to their symptoms could help in maximizing their autonomy and control. There is a need for education that would help people with PD and their family members thoroughly understand both motor and non-motor symptoms.

The third most popular topic was information related to medical systems. The hospital recommendations for treatment and diagnosis can be explained by the lack of information about specialized clinics for PD and the prevalent doctor shopping culture in Korea. In fact, an awareness survey showed that on average it takes 9.4 months for people with PD to visit a hospital from the time of symptom onset, and 5 years for 13% of all people with PD [30]. In a previous study, people with PD and their families responded that health policies for the introduction of new technology and the reduction in treatment costs are most necessary [31]. In addition, while people with PD pay just 10% of their medical expenses thanks to the relief co-payment policy for rare and intractable diseases, expenses for using respite care services and purchasing assistance devices incurred for various complications and non-motor symptoms are not covered [30]. Therefore, there is a need for policies and support that would reflect the disease characteristics of PD.

The fourth most popular topic was rehabilitation-related information. Since people with PDs’ performance of daily activities deteriorates with time due to the disease’s degenerative nature, they require continuous rehabilitation therapies and exercises to minimize dysfunctions, given that they expect to maintain their ability to perform daily activities through rehabilitation and exercise [32]. In comparison, the United Kingdom provides various rehabilitation and exercise-related information not only to people with PD but also to their healthcare providers [33]. However, practically no information about rehabilitation and services for people with PD is provided by the public healthcare system in Korea. Since maintaining the ability to perform activities of daily living through rehabilitation affects the quality of life [32], it is necessary to provide rehabilitation services and devise plans to activate their use.

This study contributes to the literature by confirming the need for education and care services that reflect the characteristics of the disease. However, it has the following limitations. First, it is difficult to generalize the data as it was collected from a single online community for people with PD and their family members, whose main users were their children, who had a relatively high level of e-health literacy. Second, since we collected anonymized data from an online patient community, the patients’ clinical or socio-demographic characteristics were not analyzed. Finally, we did not evaluate the accuracy of the information. In a future study, the information needs to consider patients’ clinical characteristics and the accuracy of the information should be explored. In addition, as unmet information needs for PD and treatment-related information were high in this study, we suggest a future study to develop and apply an educational program to increase the understanding of the disease and treatment for people with PD and their families.

## 5. Conclusions

Unmet information needs of people with PD and their family members focused more on treatment-related information, such as the effects and side effects of medications for Parkinson’s disease and surgical treatment. This treatment-related information was basic information that people with PD and their families should know from the early stages of the disease. In this study, there was a high demand for treatment-related information in the online patient community.

Many people with PD and their family members are increasingly relying on online and internet resources rather than traditional information sources. Healthcare professionals, including physicians, registered nurses, and advanced practice nurses, should be aware of and acknowledge the desire of people with PD and their family members to discuss and clarify information from online communities. As for the method of providing information for people with PD and their families, it will be effective to provide tailored education services using online communities and the internet by using their information needs and preferred resources.

## Figures and Tables

**Table 1:** Typology of unmet information needs related to PD from the free-posting messages. *N* = 1348.

Categories (*n*, %)	Subcategories	*n*	%
1. Parkinson’s Disease-specific information (*n* = 412, 30.57%)	Symptoms/management of symptoms	266	19.73
	Etiology and nature of the disease	72	5.34
	Diagnostic test or process	28	2.08
	Stage of the disease	21	1.56
	‘On-off’ phenomenon	19	1.41
	Where to get reliable information about the disease	6	0.45
2. Treatment-related information (*n* = 553, 41.01%)	Medication effects and side effects	272	20.18
	Complementary and alternative medicine	65	4.82
	Deep brain stimulation	44	3.26
	Other patients’ experiences or choices relating to treatment	36	2.67
	Decision-making on treatment options	34	2.52
	Drug interactions	32	2.37
	Oriental medicine	19	1.41
	Enteral feeding	15	1.11
	New medicines/interventions	14	1.04
	Medical cannabis	10	0.74
	Stem cell therapies	9	0.67
	Clinical trials	3	0.22
3. Rehabilitation information (*n* = 87, 6.54%)	Rehabilitation therapy	26	1.93
	Medical supplies or medical equipment	24	1.78
	Exercise	19	1.41
	Activities of daily living issues	14	1.04
	Diet	4	0.30
4. Medical system information (*n* = 169, 12.54%)	Hospital recommendations for treatment	79	5.86
	Korean national health insurance coverage	28	2.08
	Hospital recommendations for diagnosis	18	1.34
	Interaction with health care providers	17	1.26
	Experience or qualifications of physicians and medical staff	13	0.96
	Private health insurance coverage	7	0.52
	Hospital transfers	7	0.52
5. Care-related information (*n* = 43, 3.18%)	Nursing care products and equipment	15	1.11
	Long-term care facilities	12	0.89
	Day care centers	8	0.59
	Home care services	8	0.59
6. Interpersonal/social information (*n* = 71, 5.27%)	Social welfare services	43	3.19
	Caregivers’ experiences about caregiving	17	1.26
	Self-support groups	6	0.45
	Effects on social life or leisure	5	0.37
7. Financial/legal information (*n* = 13, 0.96%)	Cost of treatment, formal caregiving, or other financial issues	13	0.96

**Table 2:** Frequently asked questions relating to side effects of medication for PD. *N* = 117.

Side Effects of Medication for Parkinson’s Disease	*n*	%
Hallucinations	20	17.09
Drowsiness	17	14.53
Dizziness	14	11.97
Nausea and vomiting	12	10.26
Constipation	6	5.13
Dyskinesia	5	4.27
Orthostatic hypotension	4	3.42
Heartburn	4	3.42
Dry mouth	4	3.42
Sudden sleepiness	4	3.42
Loss of appetite	3	2.56
Skin problems	3	2.56
Delusions	3	2.56
Burning tongue	3	2.56
Compulsive behaviors	3	2.56
Headache	2	1.71
Trouble with memory or concentration	2	1.71
Hair loss	2	1.71
Sleep disturbance	1	0.85
Diplopia	1	0.85
Anxiety	1	0.85
Chilling	1	0.85
Fever	1	0.85
Dyspnea	1	0.85

**Table 3:** Frequently asked symptoms related to PD in questions. *N* = 266.

Categories (*n*, %)	Symptoms Related PD	*n*	%
Motor symptoms (*n* = 94, 35.34%)	Rigidity	23	8.65
	Dysphagia	23	8.65
	Gait disturbances	13	4.89
	Tremors	12	4.51
	Dystonia or dyskinesia	9	3.38
	Akinesia or bradykinesia	6	2.26
	Postural instability	4	1.50
	Speech problems	4	1.50
Non-motor Symptoms (*n* = 172, 64.66%)	Gastrointestinal issues	24	9.02
	Pain	23	8.65
	Sleep problems	19	7.14
	Psychosis	18	6.77
	Lightheadedness	13	4.89
	Personality changes	10	3.76
	Dyspnea	9	3.38
	Urinary difficulties	8	3.01
	Depression and anxiety	7	2.63
	Paresthesia	7	2.63
	Weight loss and loss of appetite	6	2.26
	Skin problems	6	2.26
	Cognitive changes	5	1.88
	Sweating	4	1.50
	Fatigue	3	1.13
	Eye/vision or hearing issues	3	1.13
	Leg edema	3	1.13
	Sexual concerns	1	0.38
	Fissured tongue	1	0.38
	Dry mouth	1	0.38
	Disturbances in the sense of smell	1	0.38

## Data Availability

The data that support the findings of this study are available from the corresponding author, upon reasonable request.
